# Inflammatory Pseudotumor of the Spleen in a Patient With Psoriatic Arthritis: A Diagnostic Challenge in the COVID-19 Era

**DOI:** 10.7759/cureus.73121

**Published:** 2024-11-06

**Authors:** Alexandros S Iliadis, Triantafyllia Koletsa, Periklis Vounotrypidis, Athanasios Fassas, Athanasios Apostolidis, Stylianos Apostolidis, Anastasia Fotiadou, Prodromos Hytiroglou

**Affiliations:** 1 Department of Pathology (Laboratory of General Pathology & Pathological Anatomy), Aristotle University of Thessaloniki, Thessaloniki, GRC; 2 Department of Pathology, Laboratory of Diagnostic Histopathology, Thessaloniki, GRC; 3 Department of Rheumatology, 424 General Military Hospital, Thessaloniki, GRC; 4 Department of Oncology, St. Luke's Hospital, Thessaloniki, GRC; 5 Department of First Propaedeutic Surgery, AHEPA University General Hospital of Thessaloniki, Thessaloniki, GRC

**Keywords:** covid-19, disease modifying anti-rheumatic drugs, inflammatory pseudotumor, mrna-based vaccine, psoriatic arthritis, rheumatic disease, sars-cov-2, spleen, tumor necrosis factor-α(tnf-α) inhibitor

## Abstract

The baseline inflammatory microenvironment in various organs of patients, which is shaped by pre-existing conditions and circulating drugs at the time before viral antigen exposure, may affect the severity of coronavirus disease-19 (COVID-19) infection and the nature of its complications. Inflammatory pseudotumor (IPT) of the spleen may represent one such complication that merits further investigation. We describe the case of a patient, who was under long-term treatment with a tumor necrosis factor inhibitor (TNFi), for psoriatic arthritis (PsA) and developed an inflammatory mass in the spleen, accompanied by systemic manifestations. This occurred with a history of four doses of nucleoside-modified messenger RNA (modRNA) vaccination for COVID-19 and shortly after a SARS coronavirus-2 (SARS-CoV-2) infection. Histologic examination of the splenectomy specimen supported by a large array of immunohistochemical stains and subsequent clinicopathological correlation indicated the diagnosis of splenic IPT in an immunologically altered background, due to modifying medication, immunization, and infection. Our case speculates that IPT may represent an adverse event related to immunogenicity of SARS-CoV-2, following antigen exposure (at first by sequential modRNA COVID-19 vaccinations and additionally by natural infection), despite the potentially protective effect of treatment with a TNFi.

## Introduction

Inflammatory pseudotumor (IPT) represents the unregulated, yet non-neoplastic growth of inflammatory cells, irrespective of their organ of origin, and presents serious diagnostic challenges for the pathologist [1]. It is a rare, benign cause of splenomegaly and a reactive lesion that should be distinguished from the clinicopathologically similar, but biologically distinct inflammatory myofibroblastic tumors (IMT), mostly found in soft tissues. SARS coronavirus-2 (SARS-CoV-2) infection has been characterized by a predilection to cause disruptive immune responses involving excessive inflammation and organ injury, including complications such as hyperinflammatory syndromes. Patients with underlying systemic rheumatic diseases often present an increased inflammatory set-point and a tendency to potentially develop such secondary hyperinflammatory injuries [2]. Immune system modifying agents used in patients with systemic rheumatic diseases may variably affect susceptibility to coronavirus disease-19 (COVID-19) and its associated hyperinflammatory syndromes [3]. Specific biologic disease-modifying anti-rheumatic drugs, such as tumor necrosis factor inhibitor (TNFi), have been postulated to moderate complications associated with COVID-19 hyperinflammation [4]. Golimumab (GM) is a human transgenic IgG1 tumor necrosis factor-alpha (TNFα) antagonist monoclonal antibody with the primary function of targeting and neutralizing TNFα, and so preventing inflammation as a TNFi [5]; it is approved for use in the treatment of patients with long-term immune-mediated inflammatory disorders, including, among others, psoriatic arthritis (PsA) [6]. We report a patient with long-term PsA on GM treatment for five years, who developed a sizeable IPT of the spleen, following SARS-CoV-2 infection, which had been preceded by administration of four doses of nucleoside-modified messenger (modRNA) vaccination for COVID-19. A review of the related literature reveals that this is only the third case report of IPT associated with SARS-CoV-2.

## Case presentation

**Figure 1:**
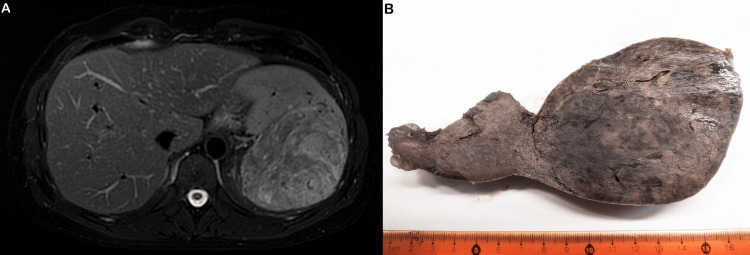
Cross-sectional imaging and gross features A. Preoperative upper abdomen magnetic resonance imaging revealing a prominent splenic mass; B. The cut surface of the surgical specimen shows a protruding spheroid mass with well-defined borders, brownish color, and solid texture.

This 42-year-old man, with a 23-year history of psoriasis vulgaris, presented in 2007 with bilateral antebrachiocarpal joint synovitis, as well as hand, elbow, and knee tendonitis. Diagnosis of PsA was made. Treatment with methotrexate (MTX) (12.5 mg/week, orally), prednisolone (10 mg→5 mg), and folic acid (5 mg/week) was started. Due to insufficient initial response leflunomide (10 mg/day) was added, which was later discontinued upon clinical improvement. Disease remission, under treatment, lasted two years. Due to a flare in 2010, adalimumab (ADA) (40 mg/15 days) was added, with gradual corticosteroid and subsequent MTX (due to parenthood plans) discontinuation, the latter being added again the following year (10 mg/week), due to recurrence of knee arthritis. In 2012 dosages of MTX (7.5 mg/week) and ADA (40 mg/20 days) were reduced. In 2014, MTX administration was interrupted again (due to parenthood plans) while sustaining ADA monotherapy (40 mg/15 days). Periodically, there was knee pain upon exertion, high inflammation markers, and diarrhoeal manifestations. Colonoscopy showed no pathological findings. Short-term treatment with colchicine was added (max. of 1 mg/day, for 15-20 days) during knee arthropathy flares. In 2015, high inflammation markers (erythrocyte sedimentation rate (ESR): 35-45 mm/hour, C-reactive protein (CRP): 3x normal) were found without clinical manifestations, and sulfasalazine (SSZ) (max. of 2 g/day) was added to the treatment, as well as probiotics during diarrhoeal episodes and chlordiazepoxide intermittently due to coexisting anxiety manifestations. After two years on ADA and SSZ with improvement of joint symptoms, SSZ treatment was terminated in 2017, and ADA monotherapy was discontinued after a new arthritis and psoriasis flare in 2018. In its stead, the patient began treatment with methylprednisolone (8 mg→2 mg) and GM (50 mg→100 mg/month), with excellent response. Non-steroidal anti-inflammatory drugs were taken on demand for short periods. The patient received the following COVID-19 modRNA vaccination scheme: three doses of vaccine BNT162b2 (Comirnaty, Pfizer-BioNTech) in 2021 and one dose of vaccine mRNA-1273 (Spikevax, Moderna) in February 2022. In late September 2022, SARS-CoV-2 infection and COVID-19 were confirmed, causing two days of fever with chills and no other symptoms consequently, except for fatigue. No other inciting events occurred. Three months later, in January 2023, the patient presented with weight loss, cachexia, and fatigue. No fever or night sweats were reported. Laboratory evaluation revealed iron deficiency anemia, elevated inflammation markers (ESR and CRP), and increased polyclonal IgG levels. Other routine biochemical and hematological tests were all within the normal ranges. Colonoscopy and MR-enteroclysis showed no pathological findings. Upper abdomen MRI revealed a large splenic mass, hypo- to iso-intense to the surrounding spleen (Figure 1A). Splenectomy was performed in 02/2023 and sent for histologic examination.

**Figure 2:**
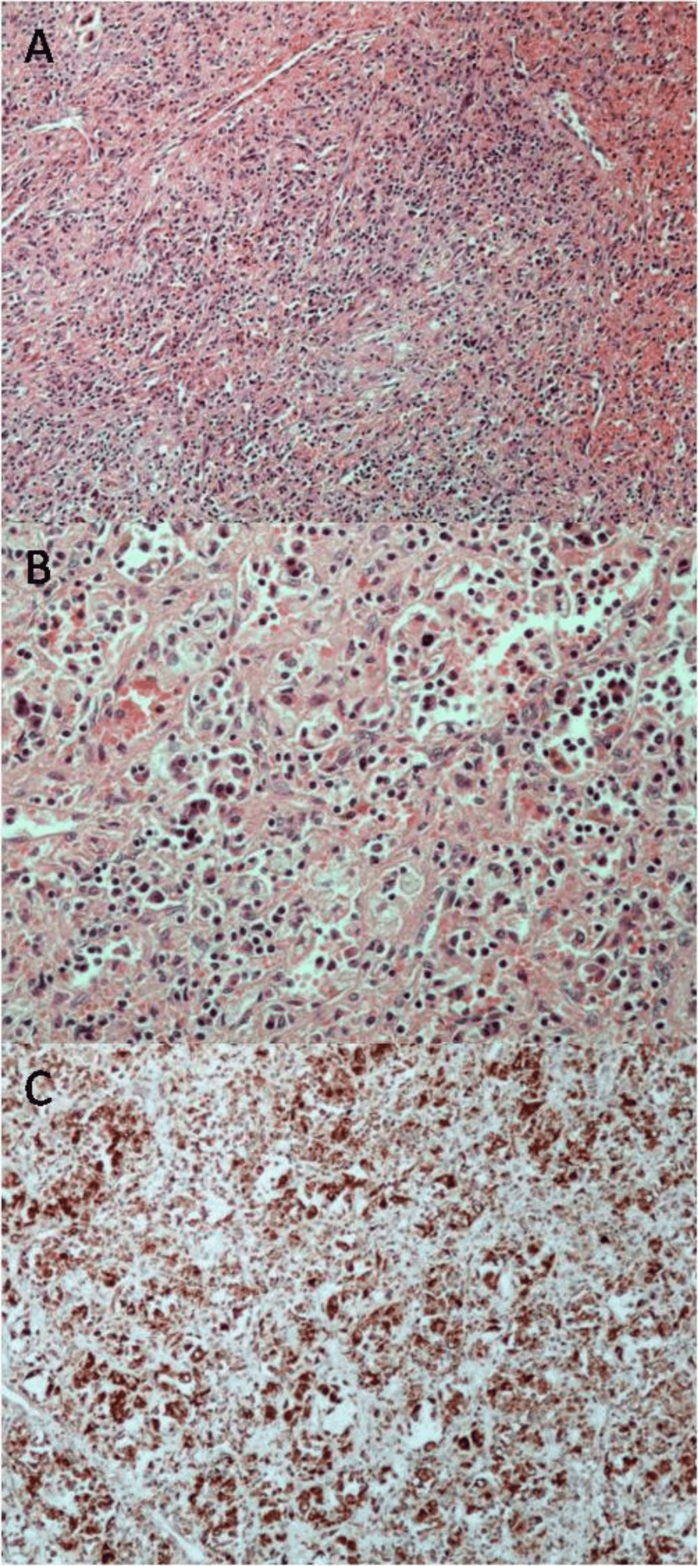
Histopathological features A, B. Red pulp substrate with fibroblastic reaction and moderate to severe inflammatory infiltrates, including "foamy" and iron-laden histiocytes, plasma cells, and lymphocytes; C. Immunohistochemical stain for CD68 highlighting histiocytes (A: hematoxylin-eosin, 100x; B: hematoxylin-eosin, 200x; C: streptavidin-biotin, 100x)

The spleen, weighing 490 g and measuring 15x10x8 cm, was deformed due to the presence of a spheroid mass, measuring 10x10x7 cm and causing a subcapsular surface bulge, with relatively well-defined borders, brownish color, and solid texture (Figure 1B). Multiple cross-sections revealed a red pulp substrate with fibroblastic reaction and moderate to severe inflammatory infiltrates, including "foamy" and iron-laden histiocytes, plasma cells, and lymphocytes (Figures 2A, 2B). Focal extramedullary hematopoiesis was also found. Sections from other sites of the spleen showed preservation of the normal architecture. Histochemical stains (PAS, Giemsa, and Ziehl-Neelsen) showed no microorganisms. Immunohistochemistry (IHC) for κ and λ light chains revealed polytypic plasma cells. Histiocytes showed CD68 positivity (Figure 2C). No neoplastic cells were found on multiple hematoxylin-eosin-stained sections and IHC for α-SMA, CD21, CD23, ALK1, CD30, CD31, CD34, and ERG. IgG4 immunostain showed few positive plasma cells. IHC for HHV-8 antigen and in-situ hybridization for Epstein-Barr virus RNA (EBER) were negative. The histological features of the splenic mass were compatible with IPT. A splenic hilar lymph node showed no appreciable pathologic alterations. Due to extramedullary hematopoiesis, a bone marrow biopsy was performed, without any evidence of neoplastic or hematological disease. Clinicopathological correlation suggested a pseudotumor possibly of infectious etiology, in an immunologically altered background due to modifying medication, immunization, and infection. Laboratory evaluation following splenectomy showed normalization of hemoglobin, IgG, and CRP levels. The patient remains asymptomatic with all laboratory values in normal range ca. 20 months post-splenectomy.

## Discussion

The term "inflammatory pseudotumor" refers to a globally benign and uncommon condition that may occur almost anywhere in the body, including the spleen, albeit rare [1]. The precise number of primary solitary splenic IPT cases is unknown, with various numbers reported in the literature since its first description in 1984, reaching estimates as high as only 116 in recent studies up until 2016. However, the obtainable data are incomplete, and it is still not explicit whether some of these cases represent secondary splenic involvement or even misdiagnoses of other histologically resemblant lesions. IPT mimics neoplastic processes raising differential diagnostic problems and has been documented under several nomenclature and histologic appearances, mainly due to pathogenetic variations described in several cases [7]. It mostly affects middle-aged to old individuals causing weight loss, pyrexia, abdominal pain, and splenomegaly. The majority of cases of splenic IPT manifest as a single, well-circumscribed, spherical mass on radiologic examination.

Current research on the pathogenesis of IPT has highlighted a complex interplay of immune dysregulation, infectious agents, and genetic alterations [7]. Studies have suggested a role for Epstein-Barr virus (EBV) [8] and other pathogens in triggering aberrant immune responses, leading to localized inflammation and tumor-like masses. IMT, a subtype of IPT, have been found to harbor ALK gene rearrangements, which are thought to drive abnormal cellular proliferation and have led to the development and application of targeted therapies, such as ALK inhibitors, for specific IPT cases. Immune system dysregulation, particularly involving macrophages and T-cells, appears to contribute to the excessive fibroinflammatory response observed in IPT. Overall, the pathogeny of IPT involves multifactorial processes and ongoing research aims to better comprehend these mechanisms to improve diagnostic and therapeutic approaches.

Histologic and immunophenotypic variability is evident among previously described cases, characterized by myofibroblastic, follicular dendritic cell differentiation, or even undifferentiated cells, leading to the speculation that perceives IPT actually as an "umbrella" category [8]. An extensive IHC panel is usually used in order to define the cell of origin or the underlying pathogenesis. In this presented case, spindle cells were negative to all markers applied in the IPT workup. Although an infectious causative agent is commonly suspected in splenic IPT [9], in our case, it was not proven, after a thorough clinical workout and broad IHC investigation.

GM is known to be rarely associated with several hematological or lymphoproliferative adverse events or side effects, as is the case with similar bDMARDs [10,11], yet not with IPT. TNFi can modify chronic inflammatory processes by restoring cytokine and immune cell balance [12]. The fact that our patient developed IPT while receiving a TNFi, for which literature has shown evidence of effective use in certain cases of IPT [13], is of particular interest, especially taking into account recent reports linking COVID-19 or modRNA COVID-19 vaccination with IPT development [14-16]. In this context, IPT has been considered to be a manifestation of delayed hypersensitivity type reaction [15]. Our patient developed symptoms that led to the discovery of the splenic IPT three months after the SARS-CoV-2 infection and almost a year after the fourth (final) dose of the modRNA COVID-19 vaccine. Therefore, the sequence of events may support an aetiologic relationship between SARS-CoV-2 infection and IPT; nevertheless, the possibility of a pathogenetic mechanism initiated by the vaccines cannot be ruled out. The main limitation of this report cautioning against generalization without additional reports would be its singularity and the lack of related data in the literature.

## Conclusions

The baseline inflammatory microenvironment in various organs of patients, which is shaped by pre-existing conditions and circulating drugs at the time before viral antigen exposure, may affect the severity of COVID-19 development and the nature of its complications. Splenic IPT may represent one such complication that merits additional investigation. Therefore, we draw the attention of clinicians, radiologists, and pathologists to the possibility of post-vaccine or post-infection IPT, even during TNFi administration, especially, but not exclusively, in patients with systemic rheumatic disease. Such lesions may be symptomatic, raising issues of differential diagnosis in patients with chronic inflammatory disorders. Reports of additional cases will help elucidate the pathogenetic mechanisms involved. In conclusion, our case suggests that IPT may represent an adverse event related to immunogenicity of SARS-CoV-2, following antigen exposure (at first by sequential modRNA COVID-19 vaccinations and additionally by natural infection), despite the potentially protective effect of treatment with TNFi.
